# A pilot randomized controlled trial of acupuncture at the *Si Guan Xue* for cancer pain

**DOI:** 10.1186/s12906-017-1838-5

**Published:** 2017-06-26

**Authors:** To-Yi Lam, Li-Ming Lu, Wai-Man Ling, Li-Zhu Lin

**Affiliations:** 1 Department of Oncology, Guangzhou University of Chinese Medicine, Guangzhou, 510405 China; 2 The Second Affiliated Hospital of Guangzhou University of Chinese Medicine, Guangdong Provincial Hospital of Chinese Medicine, Guangzhou, 510120 China; 3 Department of Clinical Oncology, Pamela Youde Nethersole Eastern Hospital, 3 Lok Man Road, Chai Wan, Hong Kong

**Keywords:** Acupuncture, *Si guan Xue*, Cancer pain, Randomized controlled trial

## Abstract

**Background:**

Pain is a common symptom in cancer patients. Acupuncture is a suggested treatment for a wide range of clinical conditions, usually for its beneficial effects on pain control. *Si guan xue* (the four points) have been widely used in clinical practice, and has shown that it is highly effective, effective in obtaining *qi*, shows strong acupuncture stimulation, and is simple to manipulate and safe to use. Therefore, the aim of this study is to test the protocol and safety of acupuncture at the *si guan xue* in the management of cancer pain.

**Methods:**

This is a single-blind, randomized controlled pilot trial. 42 patients with moderate to severe cancer pain were randomly assigned to three different arms with seven sessions of treatment; that is, treatment arm 1 (the *si guan xue* arm, *n* = 14), treatment arm 2 (the *si guan xue* plus commonly used acupoints arm, *n* = 14) and the control arm (the commonly used acupoints arm *n* = 14). Primary outcomes included acupuncture relieving cancer pain, and patients’ subjective improvement as measured by the Patient Global Impression of Change (PGIC). Secondary outcomes included the scores of the European Organization for Research and Treatment of Cancer Quality of Life Questionnaire-Core 30 (EORTC QLQ-C30) and Karnofsky’s Performance Status (KPS).

**Results:**

The analysis showed that the cancer pain reduction in treatment arm 2 was most prominent on day 5 when compared with the control arm (*P*<0.05). There was no difference in the scores of PGIC, EORTC QLQ-C30 or KPS among the three groups (*P*>0.05). Furthermore, no serious adverse events were observed.

**Conclusions:**

These results indicate that acupuncture at the *si guan xue* plus commonly used acupoints tends to be effective in reducing cancer pain. However, the sample size was small, and a future multi-centre study with a larger sample size is warranted.

**Trial registration:**

ChiCTR-IOR-15007471 (Retroactively registered on 28 NOV 2015)

## Background

Cancer is one of the world’s leading causes of death. According to recent reports, 12.7 million people diagnosed with cancer in 2008, and 7.6 million people died of it, accounting for 13% of all the deaths. It has been predicted that the total number of cancer deaths worldwide will increase from 13% in 2007 to 45% in 2030. For newly developed countries, the number is expected to increase from 11.3 million in 2007 to 15.5 million in 2030 [1].

Pain is a common cancer symptom. According to the World Health Organization (WHO) in 1995, there are 24.6 million cancer patients worldwide, 20% to 30% of whom suffer from various levels of pain. Although the three-step analgesic ladder of the WHO has been widely used, there is still a paucity of effective pain controls for many cancer patients, especially those in advanced stages of the disease. Many of these patients need to use analgesics such as opioids which act on the central nervous system. However, they may cause serious side effects, including nausea, vomiting, constipation, dryness of mouth, urinary retention, sleep disturbance, confusion, hallucinations, or even respiratory distress. They can give rise to intolerance among patients and coping difficulty for their families [2, 3].

Acupuncture is recommended for a wide range of clinical conditions. In particular, it has beneficial effects on pain control, and more importantly is safe to use. The benefits of acupuncture include rapid onset of analgesic effect, long sustained remission, ease of application, no risk of drug dependence or addiction, and by and large the absence of serious side effects [4].

*Si guan xue* is a unique combination of acupoints for acupuncture. It was first described in the "Twelve of the original nine-pin, *Lingshu.*" It pointed out that "there are five *zang* and six *fu*. The six *fu* come from twelve original *fu*. The original twelve come from the *si* (four) *guan*. The *si guan* treat the five *zang.*" Then Yang Jizhou, a famous Chinese Medicine Practitioner of the Ming Dynasty added that, "the *si guan* are *Taichong* (LR3) and *Hegu* (LI4)" [5]. The combination of these acupoints has the important effect of communicating the *yin* and *yang*, *zang* and *fu*, superior and inferior parts of the human body, and the *qi* and blood, and thus complementing each other. The *si guan xue* are widely used clinically. They have high treatment efficacy, easily get to the *qi*, are simple to manipulate, and safe to use [6, 7]. Their clinical use and efficacy for many clinical conditions are worthy of further studies. Therefore, this pilot study aims at testing the safety of acupuncture at the *si guan xue* in the management of cancer pain and informing the protocol for a larger study.

## Methods

### Settings and subjects

A pilot randomized clinical trial was conducted in the Department of Oncology at the First Affiliated Hospital of Guangzhou University of Chinese Medicine and at the OncWell Integrated Cancer Centre in Hong Kong. The trial lasted from May 2012 to February 2014. The study protocol was approved by the Ethics Committee of Hong Kong East Cluster (Reference no.: HKEC-2012-024) before the start of the study. The reporting of the study complied with the requirements of the CONSORT 2010 statement [8].

Patients who fulfilled the following criteria were recruited into this study: (1) having advanced cancer with cancer pain as the chief complaint; (2) no chemotherapy or radiotherapy within one month before the study; (3) men or women aged 18 years or above; (4) levels of conscious to the extent that he/she could evaluate and report on their pain; (5) life expectancy of three months or above; and (6) capability of giving informed consent. Those with the following conditions were excluded: (1) pain was unrelated to cancer; (2) inability to report his/her pain accurately; (3) mental illness or lack capacity; (4) children or pregnant/lactating women; (5) other serious diseases; (6) unwillingness to cooperate or give informed consent [9–13].

### Randomization and blinding

A computer program was used to randomize the participants [14]. The study coordinator was responsible for allocating the randomization codes that indicated the arms into the sequentially numbered and sealed envelopes. These envelopes were concealed from the investigators. In this pilot study, neither the investigators nor the participants were blinded, however the analyst was blinded.

### Treatment procedures

The Chinese Medicine style of acupuncture was used. Acupuncture was conducted by two qualified Chinese Medicine Practitioners with nine and twelve years of experience in clinical practice, and three and ten years of acupuncture experience respectively. Both have bachelor’s and master’s degrees in Chinese Medicine related fields (one of whose master’s degrees is in acupuncture), and are well trained in acupuncture. A course of acupuncture with seven treatment sessions, delivered either daily or on alternating days, was given to the patients in the three arms. The standards of the study complied with the requirements of the Checklist for items in the Standards for Reporting Interventions in Clinical Trials of Acupuncture (STRICTA) 2010 [15].

#### Theoretical basis for using acupuncture to treat cancer pain

The design of our acupuncture treatment protocol was based on traditional Chinese Medicine theory. Pathophysiology of cancer pain, based on Western Medicine, did not affect the formulation of acupuncture interventions in this study. According to the Chinese medical diagnosis, diseases can be conceptualized as conditions of deficiency or strength. The former refers to the insufficiency of *qi* and blood, *ying* and *yang*, *zang fu* etc., whereas the latter implies a surplus of *qi* and blood. The normal circulation of *qi* and blood in the meridians requires warming by *yang*, moisturizing by *yin*, promotion by *qi* and the nourishment by blood. Deficiency of these elements leads to the malnutrition of *zang fu,* and then the pain. Deficiency is more prominent in advanced cancer patients [16, 17]. The *si guan xue* have been considered to have an important effect on communicating the *yin* and *yang*, *zang* and *fu*, superior and inferior parts of the human body, as well as the *qi* and blood. They thus complement each other. Hence, we hypothesized that *si guan xue* would enhance the treatment effects of the commonly used acupoints.

#### The choice of Acupoints

There were two treatment arms in the study. Treatment arm 1 used only the *si guan xue*, whereas treatment arm 2 used the *si guan xue* in combination with a set of commonly used acupoints. This set of commonly used acupoints, including *Neiguan* (PC6), *Zusanli* (ST36) and *Sanyinjiao* (SP6), were chosen according to a previous extensive literature review [18–32]. They also constituted the control arm of the pilot study. All these acupoints, especially those located below the elbow and knee joints of the twelve regular meridians, have therapeutic effects on diseases in both the local and the remote regions.

#### Acupuncture intervention

Single-use acupuncture needles (0.25 x 25 mm or 0.30 x 40 mm) manufactured by MOCM International Development Limited were inserted under the skin at 10-20 mm vertical depth. Then, a reinforcing-reducing method was used to activate the *qi* until the sensation of the arrival of *qi* (numbness, fullness and heaviness) was reported by patients. Patients were maintained in supine positions with the needles left in situ for 30 min. A course of acupuncture treatment consisted of seven sessions in total, performed either daily or on alternating days. Safety precautions were conducted for the patients before each treatment session to prevent acupuncture related adverse events such as fainting, hematoma, curved needles and broken needles.

### Clinical assessment

The primary outcome of the study, the change in pain, was evaluated by two methods. Firstly, it was measured by the pain assessment form with a numeric rating scale (NRS) from 0 to 10 (0 = the best, 10 = the worst) [33]. Secondly, patients’ subjective improvement was assessed using the Patient Global Impression of Change (PGIC) [34].

Secondary outcomes included the scores determined by the European Organization for Research and Treatment of Cancer Quality of Life Questionnaire-Core 30 (EORTC QLQ-C30) [35] and the Karnofsky’s Performance Status (KPS). Three sets of EORTC QLQ-C30 were completed by the participants; that is, before the start of the study, after completing the 7th acupuncture treatment, and during the follow-up visit 2 weeks after the treatment. KPS was assessed on each follow-up visit.

### Statistical analysis

The analyses were performed on the intention-to-treat population, defined as the participants who had completed baseline assessment and at least one evaluation after treatment. A repeated measures design approach was adopted to compare the treatment outcomes (the score of cancer pain reduction, Global Health Scale, Functional Scale, Symptom Scale and KPS) over time between the three groups. A model was established for performing longitudinal data analysis to explore the intervention effect and the time effect. Among-group differences at each measure time point were further examined using Analysis of Variance (ANOVA). Categorical variables, including categorical baseline variables and incidence of adverse events, were analyzed using a Chi-square (*χ* ^*2*^) or Fisher Exact test. Statistical significance was defined as a two-tailed *P <* 0.05. Statistical analysis was performed by SPSS 20.0 (IBM SPSS Inc., Armonk, New York, USA) and SAS 9.2 software (SAS Institute Inc., Cary, USA).

## Results

### Baseline characteristics

1. General Information on the Accrued Patients

**Fig. 1:**
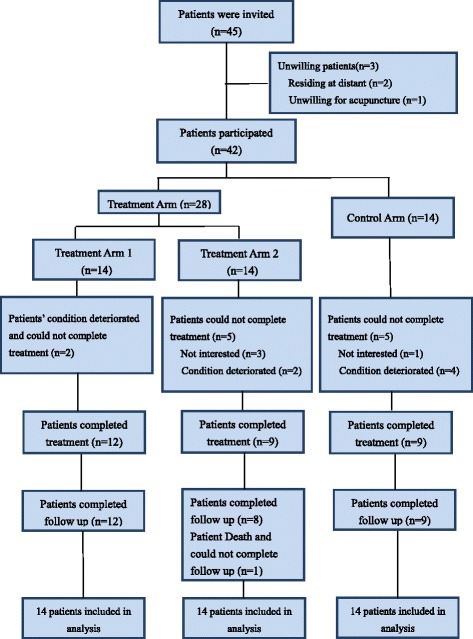
Research Flowchart

From May 2012 to February 2014, 45 cancer patients were screened. 3 refused to participate in this study because of geographical reasons or unwillingness to accept acupuncture. 42 participants (30 on the Mainland and 12 in Hong Kong) were randomly assigned to the three study arms with 14 in each of them (Fig. 1). 30 (71.4%) participants completed planned course of treatment. However, since all participants finished at least 2 sessions of treatment and at least 2 evaluations after treatment, they were all included in the data analysis.

Baseline characteristics of the patients are listed in Table 1. There were 20 men and 22 women. For patients on the Mainland, the majority had their primary cancer disease in the lung (*n* = 11), while it was in the head or neck for those in Hong Kong (*n* = 4). Most of the patients had stage IV cancer (*n* = 37), and some had been suffering from another chronic disease as well (*n* = 26) (Table 1). Before the acupuncture, the mean of pain score was 5.81, which was a moderate level of pain [33]. KPS was 70%, indicating that they could have lived independently, but could not have maintained normal activities or work [36]. Moreover, the use of analgesics was recorded for all accrued patients. There was no statistically significant difference in analgesic use among the three study arms (*P* > 0.05) (Tables 2 and 3).

**Table 1:** Baseline characteristics of patients (*n* = 45)

General Information	All patients	Participants		Unwilling participants
		Mainland China	Hong Kong	Hong Kong
Sex				
Male	20 (47.62)	15 (50)	5 (41.67)	0 (0)
Female	22 (52.38)	15 (50)	7 (58.33)	3 (100)
Age				
Average	57.59	56	59.17	78.33
Range	24–91	24–79	42–91	74–82
Marital status				
Single	2 (4.76)	0 (0)	2 (16.67)	0 (0)
Married	38 (90.48)	30 (100)	8 (66.66)	2 (67)
Widowed	2 (4.76)	0 (0)	2 (16.67)	1 (33)
Education				
Illiteracy	8 (19.05)	6 (20)	2 (16.67)	1 (33)
Primary school	13 (30.95)	12 (30)	1 (8.33)	1 (33)
Secondary school	2 (4.76)	2 (6.67)	0 (0)	0 (0)
High school	14 (33.33)	9 (30)	5 (41.67)	1 (33)
University or above	5 (11.91)	1 (3.33)	4 (33.33)	0 (0)
Primary Disease Site				
Head and neck	5 (11.9)	1 (3.33)	4 (33.33)	1 (33)
Lung	12 (28.57)	11 (36.67)	1 (8.33)	1 (33)
Breast	3 (7.14)	1 (3.33)	2 (16.67)	0 (0)
Gynecological	6 (14.29)	5 (16.67)	1 (8.33)	0 (0)
Liver	5 (11.9)	5 (16.67)	0 (0)	0 (0)
Upper GI (Esophagus, stomach and spleen)	5 (11.9)	3 (10)	2 (16.67)	0 (0)
Lower GI(Colon)	3 (7.14)	2 (6.67)	1 (8.33)	0 (0)
Other	3 (7.14)	2 (6.67)	1 (8.33)	1 (33)
Stage of cancer				
III	5 (11.9)	1 (3.33)	4 (33.33)	0 (0)
IV	37 (88.1)	29 (96.67)	8 (66.67)	3 (100)
Other chronic diseases	26 (61.9)	20 (66.67)	6 (50)	2 (67)
Level of pain				
Average	5.81	5.9	5.58	7
Range	2–8	2–8	5–8	5–8
KPS score				
Average	70	69	72.5	70
Range	40–90	40–90	60–80	60–80

**Table 2:** Use of analgesics in the three study arms

Use or not	Arm 1 (*n* = 14)	Control arm (*n* = 14)	Arm 2 (*n* = 14)	*P*-value
Yes	10	6	7	0.287
No	4	8	7

**Table 3:** Types of analgesics used in the three study arms

Drugs	Arm 1 (*n* = 14)	Control arm (*n* = 14)	Arm 2 (*n* = 14)
Morphine	0	3	2
Oxycodone	3	1	3
Fentanyl patch	3	3	4
Celecoxib	2	1	1
Tramadol	2	0	0
Carbamazepine	0	2	1
Meloxicam	0	1	0

Note:* P* = 0.513

## Information on pain patients before treatment

The number of pain sites for each participant ranged from 1 to 7. There were 90 pain sites in total. Most were located on the lower limbs (*n* = 20) (Table 4). The average NRS pain score over the previous 24 h before acupuncture was 5.62. Nonetheless, the average NRS score for the most severe pain was as high as 7.48, which was defined as “severe” according to the NRS. The occurrence of this severe NRS pain score was 4.74 on average. The average for even the slightest NRS pain score was 3.1 (Table 5). The average NRS pain score right before acupuncture was 5.8, a moderate level of pain [33].

**Table 4:** Distribution of pain sites

Pain sites	Number of sites		
	All	Mainland China	Hong Kong
Head	2	0	2
Neck and shoulder	10	2	8
Upper limb	8	1	7
Upper back	12	8	4
Lower back	10	8	2
Chest	8	5	3
Upper abdomen	7	5	2
Lower abdomen	8	5	3
Lower limb	20	11	9
Other	5	2	3

**Table 5:** NRS on the level of pain over the previous 24 h

Level of pain	All patients	Mainland China	Hong Kong
Average of most severe pain	7.48	7.87	6.5
Range	3–10	5–10	3–9
Average number of severe pain outbursts	4.74	4.3	5.83
Range	0–9	1–8	0–9
Average range of slightest pain	3.1	2.93	3.5
Range	0–8	0–7	0–8
Average pain	5.62	5.73	5.33
Range	3–8	3–8	3–8
Average current pain	5.8	5.9	5.58
Range	0–9	1–8	0–9

### Primary outcome

**Fig. 2:**
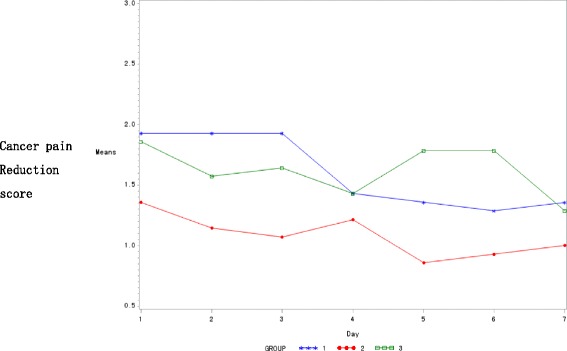
Changes of pain reduction scores in the three study arms. (Group 1: Treatment arm 1; Group 2: Control group; Group 3: Treatment Arm 2)

According to analysis of the seven treatment sessions, the magnitude of pain score reduction tended to decrease in both treatment arm 1 and control arm, while it increased in treatment arm 2 (Fig. 2). For the among-group comparisons at each time point, there was a statistically significant improvement in the pain score in treatment arm 2 on the fifth day of treatment (Table 6) (*P*<0.05). There was no difference among the three groups observed in a repeated measures design approach.

**Table 6:** Changes of NRS reduction in the three study arms $\left(\overline{x}\pm SD\right)$

Time	Arm 1 (*n* = 14)	Control arm (*n* = 14)	Arm 2 (*n* = 14)	*P*-value
Day 1	1.93 ± 1.21	1.36 ± 1.15	1.86 ± 1.03	0.36
Day 2	1.93 ± 1.14	1.14 ± 0.77	1.57 ± 1.28	0.17
Day 3	1.93 ± 1.14	1.07 ± 1.07	1.64 ± 1.22	0.14
Day 4	1.43 ± 1.16	1.21 ± 0.97	1.43 ± 1.02	0.83
Day 5	1.36 ± 1.01	0.86 ± 0.86	1.79 ± 0.89^a^	0.04
Day 6	1.29 ± 1.14	0.93 ± 0.83	1.79 ± 1.19^a^	0.11
Day 7	1.36 ± 1.08	1.00 ± 1.04	1.29 ± 1.07	0.65
Main effect of time (*P*-value)*	>0.05			

*There was no interaction between the changing trends in pain reduction scores and intervention factor of the three groups over time

^a^Compared with the control group, the difference in cancer pain reduction scores was statistically significant, *P*<0.05

On the other hand, for the patients’ perception of the overall improvement, the results of PGIC measurement across the three arms revealed that the difference in their PGIC scores was not statistically significant (*P* > 0.05) (Table 7).

**Table 7:** PGIC scores in the three study arms $\left(\overline{x}\pm SD\right)$

Time	Arm 1 (*n* = 14)	Control arm (*n* = 14)	Arm 2 (*n* = 14)	*P*-value
Day 7	2.42 ± 0.79	2.64 ± 0.67	2.56 ± 0.53	0.74

### Secondary outcomes

**Fig. 3:**
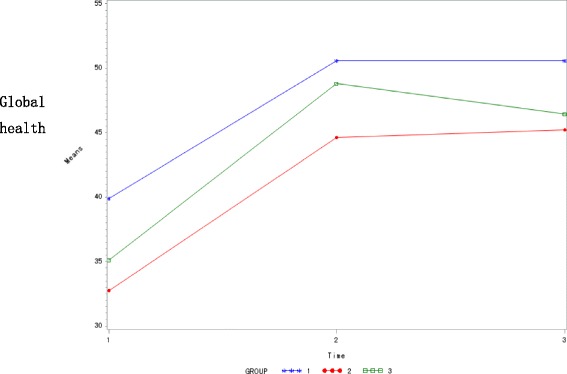
Global health scores and changes among the 3 groups. (Group 1: Treatment arm 1; Group 2: Control group; Group 3: Treatment Arm 2)

Both the Global Health Scale and the Functional Scale increased during the treatment course (Tables 8, 9 and Fig. 3), whereas the Symptom Scale decreased (Table 10). However, there was no statistically significant difference found across the three arms in all the EORTC QLQ-C30 domains (*P* > 0.05). Additionally, no difference between the three groups was observed in a repeated measures design approach.

**Table 8:** Global health score and changes in the three study arms $\left(\overline{x}\pm SD\right)$

Time	Arm 1 (*n* = 14)	Control arm (*n* = 14)	Arm 2 (*n* = 14)	*P*-value
Baseline	39.88 ± 19.39	32.74 ± 18.90	35.12 ± 15.04	0.57
Day 7	50.60 ± 17.13	44.64 ± 20.83	48.81 ± 16.62	0.68
Follow up	50.60 ± 19.47	45.24 ± 16.89	46.43 ± 18.98	0.73
Main effect of time (*P*-value)*	<0.05			

*There was no interaction between the changing trends in Global Health scores and the three groups’ intervention factors over time

**Table 9:** Functional scale score and changes in the three study arms $\left(\overline{x}\pm SD\right)$

Time	Arm 1 (*n* = 14)	Control arm (*n* = 14)	Arm 2 (*n* = 14)	*P*-value
Baseline	90.29 ± 4.00#	87.24 ± 3.89	88.60 ± 3.56	0.12
Day 7	91.56 ± 4.94	89.21 ± 4.16	90.35 ± 4.10	0.38
Follow up	90.79 ± 4.85	89.78 ± 3.53	90.48 ± 4.27	0.81
Main effect of time (*P*-value)*	<0.05			

*There was no interaction between the changing trends of Functional scale scores and the three groups’ intervention factors over time

**Table 10:** Symptom scale score and changes in the three study arms $\left(\overline{x}{x}\pm SD\right)$

Time	Arm 1 (*n* = 14)	Control arm (*n* = 14)	Arm 2 (*n* = 14)	*P*-value
Baseline	4.46 ± 1.59	5.45 ± 1.88	5.21 ± 1.86	0.32
Day 7	3.36 ± 2.39	3.93 ± 1.90	4.17 ± 1.92	0.57
Follow up	3.72 ± 2.02	3.91 ± 1.78	4.27 ± 2.10	0.28
Main effect of time (*P*-value)*	<0.05			

*There was no interaction between the changing trends of Symptom scale scores and the three groups’ intervention factors over time

**Fig. 4:**
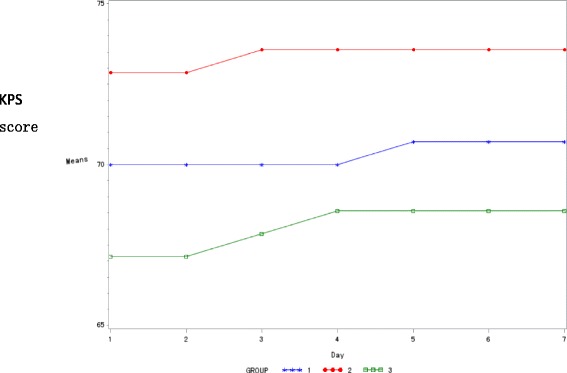
KPS score and changes among the 3 groups. (Group 1: Treatment arm 1; Group 2: Control group; Group 3: Treatment Arm 2)

The KPS score of the three arms showed an increasing trend during the treatment course, but the difference across the three arms was not statistically significant (*P*>0.05) (Table 11 and Fig. 4).

**Table 11:** KPS score and changes in the three study arms $\left(\overline{x}\pm SD\right)$

Time	Arm 1 (*n* = 14)	Control arm (*n* = 14)	Arm 2 (*n* = 14)	*P*-value
Day 1	70.00 ± 15.19	72.86 ± 13.83	67.14 ± 9.14	0.51
Day 2	70.00 ± 15.19	72.86 ± 13.83	67.14 ± 9.14	0.51
Day 3	70.00 ± 15.19	73.57 ± 13.36	67.86 ± 9.75	0.51
Day 4	70.00 ± 15.19	73.57 ± 13.36	68.57 ± 9.49	0.57
Day 5	70.71 ± 14.92	73.57 ± 13.36	68.57 ± 9.49	0.59
Day 6	70.71 ± 14.92	73.57 ± 13.36	68.57 ± 9.49	0.59
Day 7	70.71 ± 14.92	73.57 ± 13.36	68.57 ± 9.49	0.59
Main effect of time (*P*-value)*	<0.05			

*There was no interaction between the changing trends of the KPS scores and the three groups’ intervention factors over time

### Safety analysis

The safety of acupuncture, including acupuncture related fainting, hematoma, curved and broken needles was also examined in the pilot study. Throughout the study period, no serious acupuncture-related adverse events occurred. There was only one reported episode of mild local bruising, at one acupuncture site, which was successfully managed with direct digital pressure administered by the practitioner.

## Discussion

Acupuncture may be useful in controlling the pain experienced by many cancer patients. It is a complementary and conservative therapy that balances the flow of vital energy, and in turn helps to relieve pain. It is an analgesic adjunctive method for cancer patients that is worthy of additional high quality studies [37–39].

Because of the small sample size, this pilot study mainly intends to examine the preliminary effects of the acupuncture protocol using the *si guan xue* for cancer pain management for a larger study and its safety. The primary outcomes were the trend of acupuncture in relieving cancer pain and patients’ subjective improvement as measured by the PGIC. Secondary outcomes were patients’ well-being as determined by the EORTC QLQ-C30 and the KPS. According to the data analysis, the effect of pain relief tended to decrease in treatment arm 1 (*si guan xue* only) and the control arm (commonly used acupoints only). However, it increased in treatment arm 2 (a combination of *si guan xue* and commonly used acupoints), especially on the fifth day of treatment. This phenomenon reflects the fact that acupuncture treatment takes time to accumulate and exert its analgesic effect, which is consistent with the clinical experience of our routine practice [40]. On the other hand, although the trend of analgesic effect was still observed on days 6 and 7, it was not statistically significant. This may be due to the sample size of the pilot study, which is too small to detect any sustained effect.

Also, it revealed that either the acupuncture at *si guan xue* or commonly used acupoints are effective in reducing cancer pain, but that the combination of these two appears to further enhance pain reduction. Thus, this seemed to be a better acupuncture protocol for cancer pain management. There are several possible explanations for this enhanced effect. Firstly, a special technique of acupuncture was used in treatment arm 2. The *si guan xue* was opened first before regulating the commonly used acupoints. It had been recognized that this technique could produce a stronger clinical effect [41, 42]. Secondly, previous studies had suggested that acupuncture at the *si guan xue* could activate the *qi*, replenish the vital substances of the body and strengthen the visceral organs [6, 7]. Thirdly, several other studies have also supported the claim that acupuncture at the *si guan xue* could give rise to sedative, antispasmodic and analgesic effects. Thus, the use of the *si guan xue* would be more effective in controlling cancer pain [43, 44]. Though it was limited to patients with liver cancer, there has been one randomized controlled study of acupuncture at the *si guan xue*. It enrolled 86 patients and revealed that acupuncture achieved a better control on cancer pain than codeine treatment [45]. The results of our pilot study are not only consistent with these previous findings, but also reveal that the beneficial effect on cancer pain is applicable to other cancer-related diseases.

There was no statistically significant difference found across the three arms in the PGIC, EORTC QLQ-C30, and KPS scores (*P*>0.05). A possible explanation is that the treatment and evaluation period was too short to reveal the full effect of the acupuncture. However, these clinical outcomes might require longer observation times to see improvement, as documented by these scores. Therefore, the clinicians should contemplate the appropriate duration of acupuncture treatment for cancer pain. A course of 14 acupuncture sessions, or at least 2 weeks of treatment is needed for better management of this problem [40, 46]. In addition, the small sample size is another possible factor accounting for the negative results in the above outcomes.

### Limitations of the pilot study

As mentioned above, the main limitation of this study was the small sample size, which was inadequate to confirm the efficacy of acupuncture in the management of cancer pain. Moreover, the treatment and observation time were too short to reveal the full effect of the acupuncture. Seven patients even requested to continue their acupuncture treatment upon completion of the study. This may reflect the fact that acupuncture at the *si guan xue* helps to control their cancer pain and improve their quality of life, but a longer treatment course remains more desirable.

## Conclusion

The results of this pilot study indicate that acupuncture at the *si guan xue* plus commonly used acupoints tends to be effective in reducing cancer pain. It may also be beneficial in controlling cancer pain in advanced cancer patients. The use and addition of the *si guan xue* in acupuncture for cancer pain has also been found to be feasible and manageable. Although no firm conclusions can be drawn from such an under-powered study, it did show a trend towards an improvement in cancer pain scores. Therefore, further large scale and multi-centre randomized controlled trials of the *si guan xue* plus commonly used acupoints are warranted to confirm its treatment efficacy.

## Abbreviations

ANOVA: Analysis of variance
EORTC QLQ-C30: European Organization for Research and Treatment of Cancer Quality of Life Questionnaire-Core 30
KPS: Karnofsky’s Performance Status
NRS: Numeric rating scale
PGIC: Patient global impression of change
STRICTA: Standards for reporting interventions in clinical trials of acupuncture
WHO: World health organization
